# Genetic Associations of *Visfatin* Polymorphisms with EGFR Status and Clinicopathologic Characteristics in Lung Adenocarcinoma

**DOI:** 10.3390/ijerph192215172

**Published:** 2022-11-17

**Authors:** Sunny Li-Yun Chang, Po-Jen Yang, Yen-You Lin, Ya-Jing Jiang, Po-I Liu, Chang-Lun Huang, Shun-Fa Yang, Chih-Hsin Tang

**Affiliations:** 1 Graduate Institute of Biomedical Science, China Medical University, Taichung 40402, Taiwan; 2 School of Medicine, China Medical University, Taichung 40402, Taiwan; 3 School of Medicine, Chung Shan Medical University, Taichung 40201, Taiwan; 4 Department of Family and Community Medicine, Chung Shan Medical University Hospital, Taichung 40201, Taiwan; 5 Department of General Thoracic Surgery, Asia University Hospital, Taichung 41354, Taiwan; 6 Division of General Thoracic Surgery, Department of Surgery, Changhua Christian Hospital, Changhua 50006, Taiwan; 7 Institute of Medicine, Chung Shan Medical University, Taichung 40201, Taiwan; 8 Department of Medical Research, Chung Shan Medical University Hospital, Taichung 40201, Taiwan; 9 Chinese Medicine Research Center, China Medical University, Taichung 40402, Taiwan; 10 Department of Biotechnology, College of Health Science, Asia University, Taichung 41354, Taiwan

**Keywords:** *visfatin*, nicotinamide phosphoribosyltransferase, pre-B-cell colony-enhancing factor, single nucleotide polymorphism, non-small cell lung cancer

## Abstract

Lung adenocarcinoma (LUAD) is the most common histologic type of lung cancer. Mutations of the *epidermal growth factor receptor (EGFR)* gene are among the most common genetic alterations in LUAD and are the targets of EGFR tyrosine kinase inhibitors. The enzyme *visfatin* is involved in the generation of the oxidized form of nicotinamide adenine dinucleotide (NAD^+^) and regulation of intracellular adenosine triphosphate (ATP), critical processes in cancer cell survival and growth. This study explored the relationship between *visfatin* single nucleotide polymorphisms (SNPs) with EGFR status and the clinicopathologic development of LUAD in a cohort of 277 Taiwanese men and women with LUAD. Allelic discrimination of four *visfatin* SNPs rs11977021, rs61330082, rs2110385 and rs4730153 was determined using a TaqMan Allelic Discrimination assay. We observed higher prevalence rates of advanced (T3/T4) tumors and distant metastases in EGFR wild-type patients carrying the rs11977021 CT + TT and rs61330082 GA + AA genotypes, respectively, compared with patients carrying the CC and GG genotypes. EGFR wild-type patients carrying the rs11977021 CT + TT genotypes were also more likely to develop severe (stage III/IV) malignancy compared with patients carrying the CC genotype. An analysis that included all patients found that the association persisted between the rs11977021 CT + TT and rs61330082 GA + AA genotypes and the development of T3/T4 tumors compared with patients carrying the rs11977021 CC and rs61330082 GG genotypes. In conclusion, these data indicate that *visfatin* SNPs may help to predict tumor staging in LUAD, especially in patients with EGFR wild-type status.

## 1. Introduction

Lung cancer has the highest mortality rate of all cancers, according to global and national statistics in Taiwan [1,2] (Ministry of Health and Welfare, Taiwan 2021). Based on histology, lung cancer can be divided into small cell lung cancer (SCLC) and non-small cell lung cancer (NSCLC). Lung adenocarcinoma (LUAD) is the most common subtype of NSCLC and of lung cancers overall [3]. Major risk factors for lung cancer include cigarette smoking, secondhand smoking, use of domestic biomass fuels, air pollution, pulmonary conditions such as chronic obstructive pulmonary disease, and genetic factors [4].

Epidermal growth factor receptor (*EGFR*) mutations in the tyrosine kinase domain are common in NSCLC patients, with higher rates particularly in those with adenocarcinoma, non-smokers, females, and Asian populations [5,6]. Around two-thirds (67.1%) of patients with *EGFR*-mutant NSCLC have classical *EGFR* mutations (exon 21 L858R point mutation or exon 19 deletions [Ex19del]) [7]. *EGFR* mutations in the tyrosine kinase domain increase EGFR kinase activity and signs of tumorigenesis such as cancer cell proliferation, migration, invasion, and angiogenesis [8,9]. Although no molecular-targeted therapy has shown any overall survival benefit in early-stage NSCLC with *EGFR* mutations [10], advanced-stage NSCLC harboring sensitizing *EGFR*-positive mutations responds well to tyrosine kinase inhibitors (TKIs) and these agents have proven more effective than the historical standard of care, platinum-based chemotherapy [8]. In patients with untreated, *EGFR*-mutated advanced NSCLC, overall survival is improved with the third-generation EGFR TKI osimertinib compared with the first-generation TKIs gefitinib and erlotinib [11].

Given the important role of *EGFR* mutation status in NSCLC development, we decided to explore interactions amongst crucial genes and *EGFR* status in NSCLC and LUAD. Interleukin-17A, the tumor suppressor protein WW domain-containing oxidoreductase, tissue inhibitor of metalloproteinase 3 (TIMP3), and long noncoding RNA H19 polymorphisms have all been associated with clinicopathologic characteristics in lung cancers [5,9,12,13]. These four studies have helped to identify subgroups of patients at high risk for LUAD progression.

*Visfatin*, a visceral fat-derived adipocytokine, exhibits identical properties to both the pre-B-cell colony enhancing factor (PBEF) molecule that is secreted by human peripheral blood lymphocytes [14] and the enzyme nicotinamide phosphoribosyltransferase (NAMPT), and is therefore capable of synthesizing nicotinamide adenine dinucleotide (NAD^+^), a key molecule involved in the generation of adenosine triphosphate (ATP) [15]. *EGFR*-mutated NSCLC depends on a large quantity of intracellular ATP for tumor progression, so *visfatin* is critical to the survival of *EGFR*-mutated NSCLC [16].

The aberrant secretion of *visfatin* is critical for obesity-associated cancers [17,18] and has been detected in tumor and plasma samples of pancreatic ductal adenocarcinoma, oral squamous cell carcinoma (OSCC), breast cancer, renal cell carcinoma, thyroid cancer and also NSCLC [17,19]. Plasma *visfatin* levels have been correlated with tumor, node, and metastasis (TNM) staging in gastric cancer and NSCLC, and with the depth of gastric cancer invasion [17]. High plasma *visfatin* levels are also a poor prognostic factor in hepatocellular carcinoma, breast cancer, gastric cancer, and urothelial carcinoma [18]. This evidence suggests that *visfatin* could be a potentially useful marker in clinical practice for cancer diagnosis, prognosis and even for cancer therapy [15,20,21]. In SCLC, high serum *visfatin* levels are associated with brain metastases and *visfatin* appears to promote SCLC cell migration across the blood–brain barrier [22]. A recently developed dual inhibitor of *visfatin* and EGFR has shown excellent antiproliferative activities in various cancer cell lines, including H1975 NSCLC cells harboring the *EGFR^L858R/T790M^* mutation [21].

A previous study from our laboratory described how certain *visfatin* polymorphisms in a cohort of Taiwanese males were associated with higher or lower risks of developing OSCC [23]. In this study, we explored whether certain *visfatin* polymorphisms play a similar role in lung adenocarcinoma and act as potential diagnostic or therapeutic targets. Analyses specifically examined associations between four *visfatin* single nucleotide polymorphisms (SNPs) rs11977021, rs61330082, rs2110385, and rs4730153, which have been studied for their association with risk of developing various cancers [19,23,24,25], *EGFR* status and clinicopathologic characteristics in LUAD. Our results indicate that *visfatin* polymorphisms are associated with clinicopathologic staging in LUAD.

## 2. Materials and Methods

### 2.1. Patients

A total of 277 patients with LUAD harboring different *EGFR* statuses and 277 case–controls with similar baseline characteristics (Table S1) were recruited from Chung Shan Medical University Hospital, Taichung, Taiwan. Medical records from each patient were reviewed and their clinicodemographic details were recorded (age, sex, smoking behavior, tumor staging, TNM classification and cell differentiation status). Clinical disease staging was determined according to the rules in the American Joint Committee on Cancer Staging Manual. Informed written consent was obtained from each patient prior to starting the study, and the study protocol was approved by the Institute Review Board of Chung Shen Medical University Hospital (No. CS1-20144).

### 2.2. Genomic DNA Extraction and EGFR Gene Sequencing

Tumor DNA was extracted from paraffin-embedded tissues using the QIAmp DNA Mini Kit (Qiangen, Valencia, CA, USA), following the manufacturer’s protocols [26,27,28]. To classify the DNA samples as *EGFR* wild-type or *EGFR*-mutant status, L858R or exon 19 deletions (Ex19del), matrix-assisted laser desorption/ionization-time of flight mass spectrometry (MALDI-TOF MS) was used, as previously described [9].

### 2.3. Genotyping of Visfatin Polymorphisms

Peripheral blood samples were collected in ethylenediaminetetraacetic acid (EDTA) tubes and DNA was extracted using a QIAamp DNA blood mini kit (Qiagen, Valencia, CA, USA). Three of the four analyzed *visfatin* SNPs, rs11977021, rs61330082 and rs2110385, are located in the promoter region. *visfatin* rs4730153 is located at the intron region between exon six and seven. Allelic discrimination of four *visfatin* SNPs: C and T (C/T) alleles of rs11977021, G/A alleles of rs61330082, G/T alleles of rs2110385, and G/A alleles of rs4730153 was performed using the TaqMan SNP Genotyping Assay and the ABI StepOnePlus Real-Time PCR system (Applied Biosystems, Foster City, CA, USA), as previously described [23,28]. The context sequences of the four *visfatin* SNP probes on the plus (sense) strand are shown in Table S2. The results of the replication plots performed by TaqMan genotyping assay in this study are shown in Figures S1–S4.

### 2.4. Statistical Analysis

All data were analyzed for statistical significance using SAS software, version 9.1 (SAS Institute Inc., Cary, NC, USA). Differences in demographic and clinical characteristics between *EGFR* wild-type and *EGFR*-mutant patients were calculated using the Mann–Whitney *U* test and the Fisher’s exact test. Adjusted odds ratios and 95% confidence intervals (CIs) were estimated by multiple logistic regression models after controlling for age, sex, and cigarette smoking status. A *p*-value of <0.05 was regarded as statistically significant.

## 3. Results

### 3.1. Baseline Characteristics of the Study Participants

This study recruited 277 Taiwanese men and women with LUAD who were categorized as either *EGFR* wild-type (*n* = 111) or *EGFR* mutation-positive (*n* = 166) and evaluated by age, sex, smoking history, LUAD stage and grade (Table 1). The mean age was 65.36 ± 13.42 years in the *EGFR* wild-type group and 65.90 ± 13.64 years in the *EGFR* mutation group, with no statistically significant between-group difference (Table 1). Significantly higher proportions of *EGFR*-mutant patients were female versus male (64.5% vs. 35.5%, *p <* 0.001) and never-smokers versus ever-smokers (77.1% vs. 22.9%, *p <* 0.001) (Table 1). Tumor stage and TNM status did not differ significantly between the *EGFR* wild-type and *EGFR*-mutant groups. However, a comparison of tumor grade revealed that compared with the EGFR-mutant patients, the *EGFR* wild-type patients had a higher prevalence of poor cell differentiation (20.7% vs. 6.0%, *p* = 0.001) and lower rates of well-differentiated cells (7.2% vs. 11.4%, respectively, *p* = 0.001) and moderately differentiated cells (72.1% vs. 82.5%, *p* = 0.001) (Table 1).

### 3.2. No Association of Visfatin SNP (rs11977021, rs61330082, rs2110385, and rs4730153) Distribution Frequency with EGFR Status or LUAD

To examine the potential association of the *visfatin* SNPs with LUAD, the genotype frequency of the four *visfatin* SNPs in 277 LUAD patients were compared with 277 case–controls. After adjusting for age, sex, and cigarette smoking status using multiple logistic regression models, none of the genotypes for the four *visfatin* SNPs were associated with LUAD (Table 2).

To examine potential associations between *visfatin* SNPs and *EGFR* mutation status, the frequencies of four *visfatin* SNP genotypes (rs11977021 CT, rs61330082 GA, rs2110385 GT and rs4730153 GA) were compared with *EGFR* status (Table 3). After adjusting for age, sex, and cigarette smoking status, none of the four *visfatin* SNPs were associated with a statistically higher prevalence of either *EGFR* wild-type or *EGFR* mutation status (Table 3).

### 3.3. Associations between Polymorphic Genotypes of visfatin rs11977021 with Clinicopathologic Characteristics and EGFR Status

Clinicopathologic characteristics of LUAD patients stratified by *visfatin* rs11977021 genotypes are shown in Table 4. In *EGFR*-mutant patients, LUAD stage and grade did not differ significantly when comparing the CT + TT and CC genotypes. In contrast, among *EGFR* wild-type patients, the CT + TT genotype was significantly more frequent than the CC genotype in patients with stage III/IV malignancies (81.5% vs. 63.3%, *p* = 0.045), advanced (T3/T4) tumors (53.1% vs. 26.7%, *p* = 0.013), and in those with distal metastases (58.0% vs. 33.3%, *p* = 0.021). The significant difference in tumor status between the CT + TT and CC genotypes in *EGFR* wild-type patients was also observed in an evaluation of all patients, in which 43.9% with the CT + TT genotype had more advanced (T3/T4) tumors compared with 27.8% with the CC genotype (*p* = 0.016).

### 3.4. Associations between Polymorphic Genotypes of visfatin rs61330082, Clinicopathologic Characteristics and EGFR Status

Clinicopathologic characteristics of LUAD patients stratified by *visfatin* rs61330082 genotypes are shown in Table 5. In *EGFR*-mutant patients, LUAD stage and grade did not differ significantly in a comparison of the GA + AA genotype with the GG genotype. Among *EGFR* wild-type patients, the GA + AA genotype was significantly more common than the GG genotype in patients with advanced (T3/T4) tumors (53.0% vs. 25.0%, *p* = 0.010) and in those with distal metastases (57.8% vs. 32.1%, *p* = 0.019). The significant difference in tumor status between the GA + AA and GG genotypes with *EGFR* wild-type status was also observed when all patients were analyzed, as 43.7% of those with the GA + AA genotype had advanced (T3/T4) tumors compared with 28.2% with the GG genotype (*p* = 0.021). There was no association between polymorphic genotypes of *visfatin* rs2110385 or rs4730153 with clinicopathological characteristics and *EGFR* status in LUAD patients (Tables S3 and S4).

## 4. Discussion

Our observation that *EGFR* mutations are more common in females than males and in never-smokers than in ever-smokers among patients with LUAD is consistent with previous reports [6,29]. We also found that *EGFR* wild-type disease was more likely to exhibit poor cell differentiation and lower rates of well or moderate cell differentiation compared to *EGFR*-mutant disease, which is consistent with previous reports [12,13].

In this study, the four *visfatin* SNP rs11977021, rs61330082, rs2110385 and rs4730153, had no association with *EGFR* wild-type or *EGFR*-mutant status. However, when analyzing *visfatin* rs11977021 and rs61330082 genotypes in relation to clinicopathologic characteristics, the rates of the rs11977021 CT + TT and rs61330082 GA + AA genotypes were higher than those of the CC and GG genotypes, respectively, in *EGFR* wild-type patients with advanced (T3/T4) tumors and those with distal metastases. Among *EGFR* wild-type patients, having the CT + TT genotypes at rs11977021 was associated with more severe (stage III/IV) malignancy compared with having the CC genotype at rs11977021. In an analysis of the total study population, advanced (T3/T4) tumors were found in individuals with the SNP rs11977021 carrying the CT + TT genotypes compared with those carrying the CC genotype and in those with the SNP rs61330082 and the GA + AA genotypes compared with the GG genotype.

The rs11977021 and rs61330082 SNPs are located at the promoter region of *visfatin* and have been documented to affect the transcription activity of *visfatin* [30]. Among the four studied *visfatin* SNPs, rs11977021 was predicted to be a potential methylation site after analysis using NmSEER V2.0 online software (http://www.rnanut.net/nmseer-v2/, accessed on 31 August 2022) [31]. Further studies are needed to clarify whether specific genotypes at this SNP affect *visfatin*’s methylation status and expression level.

In our study, neither *visfatin* SNPs rs4730153 nor rs2110385 were associated with particular clinicopathologic characteristics in LUAD. However, our previous study reported that the SNP rs4730153 GA genotype was associated with lymph node metastasis in OSCC betel nut chewers, whereas the SNPs rs2110385 and rs61330082 had no relation to OSCC [23].

The *visfatin* SNP rs11977021 has been investigated in other cancers. Our previous investigation found that having the *visfatin* SNP rs11977021 and the CT + TT genotypes was associated with a lower risk of developing OSCC compared with those with the CC genotype at the same SNP [23]. In contrast, in another study involving patients with hepatitis B virus (HBV) infection, there was no significant association between *visfatin* rs11977021 and the risk of developing HBV-related hepatocellular carcinoma (HBV-HCC) [24].

In this study, our analysis of the G/A alleles of *visfatin* rs61330082 SNP revealed that the GA + AA genotypes was more likely than the GG to be associated with severe disease characteristics in LUAD. The GA + AA and GG genotypes in our study are equivalent to the CT + TT and CC genotypes, respectively, described in the studies referenced in this paragraph since they refer to the genotypes on the minus (antisense) strand [6,19,24,25,32] Previous analyses of *visfatin* SNP rs61330082 have reported that it is associated with an increased risk of NSCLC, esophageal squamous cell cancer (ESCC), bladder cancer, and HCC [6,19,24,25,32]. In patients with NSCLC, the CT genotype, TT genotype and T allele of *visfatin* SNP rs61330082 apparently reduced the risk of NSCLC pathogenesis, whereas the CC genotype appeared to increase the risk [19]. Similarly, the CC genotype and C allele of *visfatin* SNP rs61330082 were associated with increased risk of ESCC [25].and bladder cancer, especially in smokers [32].

By contrast, in HBV-HCC, the TT genotype of *visfatin* rs61330082 was associated with a higher risk of HBV-HCC than the CC and CC + TT genotypes, but only in patients of Zhuang but not Han ethnicity [24]. Thus, it appears that *visfatin* rs61330082 polymorphisms in cancer development vary among different tumor tissues. Further studies are required to clarify the role of *visfatin* rs61330082 polymorphisms in LUAD tumorigenesis and progression.

The Allele Frequency Aggregator’s analysis of the National Center for Biotechnology Information (NCBI) database of Genotypes and Phenotypes (dbGaP) shows that for the four *visfatin* SNPs presented in this study, different ethnicities exhibit varying allele frequencies (Table S5). For *visfatin* rs61330082, the incidence of the A allele was 49.3% in Asians which was much higher than the rates in the global population and in Europeans (21.1% and 24.3%, respectively) (Table S5). This suggests Asians are more likely to have the AA + GA genotype compared with the GG genotype, the former being associated in our study with higher risk of T3/T4 tumors among all patients. Similar results were found for *visfatin* rs11977021, where the incidence of the T allele was 53.6% in Asians which was much higher than the rates in the global population and in Europeans (24.9% and 24.4%, respectively) (Table S5). This suggests Asians are more likely to have the CT + TT genotype compared with the CC genotype, the former being associated in our study with higher risk of T3/T4 tumor status among all patients.

Elevated *visfatin* levels have been detected in tumor and plasma samples in many cancers, including NSCLC [17,19]. One potential mechanism for increased *visfatin* levels may be due to specific genotypes in rs11977021 and rs61330082 resulting in increased transcription activity, although this has only been reported in obese children [30,33]. *Visfatin* is critical in NSCLC, due to its ability to increase intracellular ATP and NAD^+^ levels [16]. ATP is required to enhance the activity of receptor tyrosine kinases such as EGFR, which are in turn involved in signaling pathways needed for the survival and growth of NSCLC cells, while NAD^+^ is a substrate for enzymes such as poly (ADP-ribose) polymerase-1 and sirtuin that contribute to apoptosis resistance and tumor cell survival [16].

*Visfatin* (NAMPT) plays a pivotal role in LUAD cell survival, with NAMPT inhibition via NAMPT-small interfering RNA (siRNA) or the NAMPT inhibitor FK866 reducing the proliferation of three LUAD cell lines in one study [16], while FK866 also reduced intracellular ATP levels, dephosphorylated EGFR signal proteins and promoted apoptosis in the H1975 cell line [16]. *Visfatin* inhibitors work because cancer cells are more sensitive to *visfatin* inhibition than normal cells due to their reliance on NAD^+^ and the cancer cells are more dependent on NAD-mediated processes [21]. Normal cells, however, can synthesize NAD through an alternative pathway, i.e., nicotinic acid phosphoribosyltransferase (NaPRTase) and thereby protect themselves from *visfatin* inhibition [21].

## 5. Conclusions

Obesity is a risk factor for many cancers [34]. Although this may not appear to be the case in LUAD, which is inversely associated with high body mass index, a meta-analysis of prospective cohort studies that examined the association between measures of abdominal obesity and risk of lung cancer found that in lung cancer generally, abdominal obesity is a better predictor of malignancy than general obesity [35]. Furthermore, since *visfatin* expression is particularly enriched in visceral fat, abdominal obesity could be a possible source of elevated serum *visfatin* levels in NSCLC patients [19,36]. Indeed, higher *visfatin* levels have been found in obese individuals compared with those in nonobese controls [37,38]. Thus, it would appear that while NSCLC cells likely need to produce *visfatin* to survive, any extra *visfatin* secreted from adipose tissue would only serve to improve the tumor microenvironment.

## Figures and Tables

**Table 1:** Demographics, clinical characteristics and *EGFR* status of 277 lung adenocarcinoma patients.

Variable	*EGFR* Wild-Type (*n* = 111) n (%)	*EGFR* Mutation (*n* = 166) n (%)	*p* Value
Age			
Mean ± SD	65.36 ± 13.42	65.90 ± 13.64	*p* = 0.420
Sex, *n* (%)			
Male	67 (60.4%)	59 (35.5%)	*p* < 0.001
Female	44 (39.6%)	107 (64.5%)	
Cigarette smoking status, *n* (%)			
Never-smoker	50 (45.0%)	128 (77.1%)	*p* < 0.001
Ever-smoker	61 (55.0%)	38 (22.9%)	
Stage, *n* (%)			
I/II	26 (23.4%)	46 (27.7%)	*p* = 0.425
III/IV	85 (76.6%)	120 (72.3%)	
Tumor status, *n* (%)			
T1/T2	60 (54.1%)	107 (64.5%)	*p* = 0.083
T3/T4	51 (45.9%)	59 (35.5%)	
Lymph node status, *n* (%)			
Negative	29 (26.1%)	54 (32.5%)	*p* = 0.254
Positive	82 (73.9%)	112 (67.5%)	
Distant metastases, *n* (%)			
Negative	54 (48.6%)	78 (47.0%)	*p* = 0.786
Positive	57 (51.4%)	88 (53.0%)	
Cell differentiation, *n* (%)			
Well	8 (7.2%)	19 (11.4%)	*p* = 0.001
Moderate	80 (72.1%)	137 (82.5%)	
Poor	23 (20.7%)	10 (6.0%)	

Abbreviation: EGFR, epidermal growth factor receptor.

**Table 2:** Adjusted odds ratios (ORs) with their 95% confidence intervals (CIs) for lung adenocarcinoma associated with *Visfatin* genotype frequencies.

Genotypes	Control (*n* = 277)	LUAD (*n* = 277)	AOR (95% CI)	*p* Value
rs11977021				
CC	71 (25.6%)	72 (26.0%)	1.000 (reference)	
CT	130 (46.9%)	133 (48.0%)	1.022 (0.527–1.980)	0.950
TT	76 (27.5%)	72 (26.0%)	0.672 (0.309–1.458)	0.314
CT + TT	206 (74.4%)	205 (74.0%)	0.888 (0.475–1.659)	0.709
rs61330082				
GG	69 (24.9%)	71 (25.6%)	1.000 (reference)	
GA	131 (47.3%)	129 (46.6%)	0.926 (0.473–1.813)	0.822
AA	77 (27.8%)	77 (27.8%)	0.676 (0.313–1.461)	0.320
GA + AA	208 (75.1%)	206 (74.4%)	0.829 (0.440–1.562)	0.563
rs2110385				
GG	225 (81.2%)	228 (82.3%)	1.000 (reference)	
GT	52 (18.8%)	45 (16.2%)	0.570 (0.259–1.254)	0.162
TT	0 (0.0%)	4 (1.4%)	-	-
GT + TT	52 (18.8%)	49 (17.7%)	0.637 (0.299–1.356)	0.242
rs4730153				
GG	223 (80.5%)	230 (83.0%)	1.000 (reference)	
GA	54 (19.5%)	44 (15.9%)	0.630 (0.297–1.336)	0.228
AA	0 (0.0%)	3 (1.1%)	-	-
GA + AA	54 (19.5%)	47 (17.0%)	0.667 (0.319–1.393)	0.281

The AORs with 95% CIs were estimated by multiple logistic regression models after controlling for age, gender and cigarette smoking status. Abbreviations: AOR, adjusted odds ratio; CI, confidence interval; LUAD, lung adenocarcinoma.

**Table 3:** Distribution frequency of *visfatin* genotypes and multiple logistic regression analysis of *EGFR* mutation status in lung adenocarcinoma patients.

*Visfatin* Genotypes	*EGFR* Wild-Type (*n* = 111)	*EGFR* Mutation (*n* = 166)	AOR (95% CI)	*p* Value
rs11977021				
CC	30 (27.0%)	42 (25.3%)	1.000 (reference)	
CT	54 (48.6%)	79 (47.6%)	1.126 (0.608–2.088)	0.706
TT	27 (24.4%)	45 (27.1%)	1.247 (0.613–2.535)	0.542
CT + TT	81 (73.0%)	124 (74.7%)	1.080 (0.809–1.442)	0.601
rs61330082				
GG	28 (25.2%)	43 (25.9%)	1.000 (reference)	
GA	54 (48.6%)	75 (45.2%)	0.992 (0.531–1.857)	0.981
AA	29 (26.2%)	48 (28.9%)	1.116 (0.554–2.250)	0.759
GA + AA	83 (74.8%)	123 (74.1%)	1.018 (0.760–1.363)	0.904
rs2110385				
GG	93 (83.8%)	135 (81.3%)	1.000 (reference)	
GT	17 (15.3%)	28 (16.9%)	1.065 (0.530–2.140)	0.859
TT	1 (0.9%)	3 (1.8%)	3.833 (0.370–39.768)	0.260
GT + TT	18 (16.2%)	31 (18.7%)	1.089 (0.777–1.527)	0.619
rs4730153				
GG	93 (83.8%)	137 (82.5%)	1.000 (reference)	
GA	17 (15.3%)	27 (16.3%)	1.027 (0.509–2.072)	0.941
AA	1 (0.9%)	2 (1.2%)	3.241 (0.281–37.379)	0.346
GA + AA	18 (16.2%)	29 (17.5%)	1.057 (0.751–1.487)	0.751

AORs with 95% CIs were estimated using multiple logistic regression models after controlling for age, sex, and cigarette smoking status. Abbreviations: AOR, adjusted odds ratio; CI, confidence interval; EGFR, epidermal growth factor receptor.

**Table 4:** Clinicopathologic characteristics of lung adenocarcinoma patients stratified by *EGFR* status and genotypes at *visfatin* rs11977021.

Variable	All (N = 277)			*EGFR* Wild-Type (N = 111)			*EGFR* Mutation (N = 166)		
	CC (*n* = 72)	CT + TT (*n* = 205)	*p* Value	CC (*n* = 30)	CT + TT (*n* = 81)	*p* Value	CC (*n* = 42)	CT + TT (*n* = 124)	*p* Value
Stage									
I/II	21 (29.2%)	51 (24.9%)	*p* = 0.475	11 (36.7%)	15 (18.5%)	*p* = 0.045 ^b^	10 (23.8%)	36 (29.0%)	*p* = 0.513
III/IV	51 (70.8%)	154 (75.1%)		19 (63.3%)	66 (81.5%)		32 (76.2%)	88 (71.0%)	
Tumor status									
T1/T2	52 (72.2%)	115 (56.1%)	*p* = 0.016 ^a^	22 (73.3%)	38 (46.9%)	*p* = 0.013 ^c^	30 (71.4%)	77 (62.1%)	*p* = 0.275
T3/T4	20 (27.8%)	90 (43.9%)		8 (26.7%)	43 (53.1%)		12 (28.6%)	47 (37.9%)	
Lymph node status									
Negative	22 (30.6%)	61 (29.8%)	*p* = 0.899	10 (33.3%)	19 (23.5%)	*p* = 0.293	12 (28.6%)	42 (33.9%)	*p* = 0.526
Positive	50 (69.4%)	144 (70.2%)		20 (66.7%)	62 (76.5%)		30 (71.4%)	82 (66.1%)	
Distant metastases									
Negative	41 (56.9%)	91 (44.4%)	*p* = 0.067	20 (66.7%)	34 (42.0%)	*p* = 0.021 ^d^	21 (50.0%)	57 (46.0%)	*p* = 0.651
Positive	31 (43.1%)	114 (55.6%)		10 (33.3%)	47 (58.0%)		21 (50.0%)	67 (54.0%)	
Cell differentiation									
Well/Moderate	65 (90.3%)	179 (87.3%)	*p* = 0.505	25 (83.3%)	63 (77.8%)	*p* = 0.521	40 (95.2%)	116 (93.5%)	*p* = 0.691
Poor	7 (9.7%)	26 (12.7%)		5 (16.7%)	18 (22.2%)		2 (4.8%)	8 (6.5%)	

^a^ OR (95% CI): 2.035 (1.134–3.652); ^b^ OR (95% CI): 2.547 (1.005–6.459); ^c^ OR (95% CI): 3.112 (1.241–7.804); ^d^ OR (95% CI): 2.765 (1.149–6.652). Abbreviation: EGFR, epidermal growth factor receptor.

**Table 5:** Clinicopathologic characteristics of lung adenocarcinoma patients stratified by *EGFR* status and genotypes at *visfatin* rs61330082.

Variable	All (N = 277)			*EGFR* Wild-Type (N = 111)			*EGFR* Mutation (N = 166)		
	GG (*n* = 71)	GA + AA (*n* = 206)	*p* Value	GG (*n* = 28)	GA + AA (*n* = 83)	*p* Value	GG (*n* = 43)	GA + AA (*n* = 123)	*p* Value
Stage									
I/II	20 (28.2%)	52 (25.2%)	*p* = 0.628	10 (35.7%)	16 (19.3%)	*p* = 0.076	10 (23.3%)	36 (29.3%)	*p* = 0.448
III/IV	51 (71.8%)	154 (74.8%)		18 (64.3%)	67 (80.7%)		33 (76.7%)	87 (70.7%)	
Tumor status									
T1/T2	51 (71.8%)	116 (56.3%)	*p* = 0.021 ^a^	21 (75.0%)	39 (47.0%)	*p* = 0.010 ^b^	30 (69.8%)	77 (62.6%)	*p* = 0.398
T3/T4	20 (28.2%)	90 (43.7%)		7 (25.0%)	44 (53.0%)		13 (30.2%)	46 (37.4%)	
Lymph node status									
Negative	21 (29.6%)	62 (30.1%)	*p* = 0.934	9 (32.1%)	20 (24.1%)	*p* = 0.402	12 (27.9%)	42 (34.1%)	*p* = 0.452
Positive	50 (70.4%)	144 (69.9%)		19 (67.9%)	63 (75.9%)		31 (72.1%)	81 (65.9%)	
Distant metastases									
Negative	40 (56.3%)	92 (44.7%)	*p* = 0.089	19 (67.9%)	35 (42.2%)	*p* = 0.019 ^c^	21 (48.8%)	57 (46.3%)	*p* = 0.778
Positive	31 (43.7%)	114 (55.3%)		9 (32.1%)	48 (57.8%)		22 (51.2%)	66 (53.7%)	
Celldifferentiation									
Well/Moderate	65 (91.5%)	179 (86.9%)	*p* = 0.296	25 (89.3%)	63 (75.9%)	*p* = 0.131	40 (93.0%)	116 (94.3%)	*p* = 0.760
Poor	6 (8.5%)	27 (13.1%)		3 (10.7%)	20 (24.1%)		3 (7.0%)	7 (5.7%)	

^a ^OR (95% CI): 1.978 (1.101–3.554); ^b^ OR (95% CI): 3.385 (1.299–8.821); ^c^ OR (95% CI): 2.895 (1.171–7.156). Abbreviation: EGFR, epidermal growth factor receptor.

## Data Availability

The data presented in this study are available on request from the corresponding authors.

## Supplementary Materials

The following supporting information can be downloaded at: https://www.mdpi.com/article/10.3390/ijerph192215172/s1, Figure S1: Representative TaqMan assay for *visfatin* rs11977021 genotyping; Figure S2: Representative TaqMan assay for *visfatin* rs61330082 genotyping; Figure S3: Representative TaqMan assay for *visfatin* rs2110385 genotyping; Figure S4: Representative TaqMan assay for *visfatin* rs4730153 genotyping; Table S1: Demographics and clinical characteristics of 277 controls and 277 patients with lung adenocarcinoma; Table S2: The context sequences of the four *visfatin* SNPs in this study; Table S3: Clinicopathologic characteristics of lung adenocarcinoma patients stratified by EGFR status and genotypes at *visfatin* rs2110385; Table S4: Clinicopathologic characteristics of lung adenocarcinoma patients stratified by EGFR status and genotypes at *visfatin* rs4730153; Table S5: ALFA allele frequencies of *visfatin* SNPs.
