# Supplementary material for: Genetic Associations of Visfatin Polymorphisms with EGFR Status and Clinicopathologic Characteristics in Lung Adenocarcinoma
Source: Int J Environ Res Public Health. 2022 Nov 17;19(22):15172. doi: 10.3390/ijerph192215172 (PMC9690642; doi:10.3390/ijerph192215172)
Supplement: Supplementary file 1 [file ijerph-19-15172-s001.zip › ijerph-1998963-supplementary.pdf]

Table S1. Demographics and clinical characteristics of 277 controls and 277 patients with lung adenocarcinoma.

**Variable**	**Control** (*n*=277) **n (%)**	**LUAD** (*n*=277) **n (%)**	***p* value**
Age			
Mean ± SD	64.53 ± 4.17	65.90 ± 13.64	*p*=0.176
Gender			
Male	126 (45.5%)	126 (45.5%)	*p*=1.000
Female	151 (54.5%)	151 (54.5%)	
Cigarette smoking status			
Never-smoker	215 (77.6%)	178 (64.3%)	*p*=0.001
Ever-smoker	62 (22.4%)	99 (35.7%)	
EGFR mutation status			
Wild type		111 (40.1%)	
Mutation		166 (59.9%)	
stage			
I+II		72 (26.0%)	
III+IV		205 (74.0%)	
Tumor T status			
T1+T2		167 (60.3%)	
T3+T4		110 (39.7%)	
Lymph node status			
Negative		83 (30.0%)	
Positive		194 (70.0%)	
Distant metastases			
Negative		132 (47.7%)	
Positive		145 (52.3%)	
Cell differentiation			
Well		27 (9.7%)	
Moderate		217 (78.3%)	
Poor		33 (11.9%)	

Abbreviation: LUAD, lung adenocarcinoma.

Table S2. The context sequences of the four *visfatin* SNPs in this study.

Variable	TaqMan Assay ID	Context Sequence
visfatin (rs11977021)	C__11405260_10	ATACTCCAATCTGACCTGATTTGAC**[C/T]**TCAG TAAAAAACACTGGTGAAGTAG
visfatin (rs61330082)	C__88870749_10	ATCATGGAAGTGGAAGGTATCACCA**[G/A]**GCA CTCACCAATGTAGTAAATACTA
visfatin (rs2110385)	C__16125685_10	ATAAGACTGGTTTTCTTGAGGAAGC**[G/T]**GAAC ATGAAAGGCTATATGTCTGAT
visfatin (rs4730153)	C__2673294_10	CCTTTAATCATATCTCATGTTTACT**[A/G]**TACCA AACCATCAACATCACAGAGA

Table S3. Clinicopathologic characteristics of lung adenocarcinoma patients stratified by EGFR status and genotypes at *visfatin* rs2110385.

Variable	ALL (N=277)			*EGFR* wild type (N=111)			*EGFR* mutation (N=166)		
	GG (*n*=228)	GT+TT (*n*=49)	*p* value	GG (*n*=93)	GT+TT (*n*=18)	*p* value	GG (*n* =135)	GT+TT (*n*=31)	*p* value
Stages									
I+II	58 (25.4%)	14 (28.6%)	*p*=0.650	22 (23.7%)	4 (22.2%)	*p*=0.895	36 (26.7%)	10 (32.3%)	*p*=0.530
III+IV	170 (74.6%)	35 (71.4%)		71 (76.3%)	14 (77.8%)		99 (73.3%)	21 (67.7%)	
Tumor T status									
T1+T2	138 (60.5%)	29 (59.2%)	*p*=0.862	52 (55.9%)	8 (44.4%)	*p*=0.371	86 (63.7%)	21 (67.7%)	*p*=0.672
T3+T4	90 (39.5%)	20 (40.8%)		41 (44.1%)	10 (55.6%)		49 (36.3%)	10 (32.3%)	
Lymph node status									
Negative	69 (30.3%)	14 (28.6%)	*p*=0.815	25 (26.9%)	4 (22.2%)	*p*=0.680	44 (32.6%)	10 (32.3%)	*p*=0.971
Positive	159 (69.7%)	35 (71.4%)		68 (73.1%)	14 (77.8%)		91 (67.4%)	21 (67.7%)	
Distant metastases									
Negative	110 (48.2%)	22 (44.9%)	*p*=0.670	45 (48.4%)	9 (50.0%)	*p*=0.900	65 (48.1%)	13 (41.9%)	*p*=0.532
Positive	118 (51.8%)	27 (55.1%)		48 (51.6%)	9 (50.0%)		70 (51.9%)	18 (58.1%)	
Cell differentiation									
Well/ Moderate	200 (87.7%)	44 (89.8%)	*p*=0.684	73 (78.5%)	15 (83.3%)	*p*=0.643	127 (94.1%)	29 (93.5%)	*p*=0.912
Poor	28 (12.3%)	5 (10.2%)		20 (21.5%)	3 (16.7%)		8 (5.9%)	2 (6.5%)	

Abbreviation: EGFR, epidermal growth factor receptor.

Table S4. Clinicopathologic characteristics of lung adenocarcinoma patients stratified by EGFR status and genotypes at Visfatin rs4730153.

**Variable**	**ALL (N=277)**			***EGFR* wild type (N=111)**			***EGFR* mutation (N=166)**		
	**GG (*n*=230)**	**GA+AA (*n*=47)**	***p* value**	**GG (*n* =93)**	**GA+AA (*n* =18)**	***p* value**	**GG (*n* =137)**	**GA+AA (*n* =29)**	***p* value**
Stages									
I+II	61 (26.5%)	11 (23.4%)	*p*=0.657	22 (23.7%)	4 (22.2%)	*p*=0.895	39 (28.5%)	7 (24.1%)	*p*=0.636
III+IV	169 (73.5%)	36 (76.6%)		71 (76.3%)	14 (77.8%)		98 (71.5%)	22 (75.9%)	
Tumor T status									
T1+T2	141 (61.3%)	26 (55.3%)	*p*=0.445	52 (55.9%)	8 (44.4%)	*p*=0.371	89 (65.0%)	18 (62.1%)	*p*=0.767
T3+T4	89 (38.7%)	21 (44.7%)		41 (44.1%)	10 (55.6%)		48 (35.0%)	11 (37.9%)	
Lymph node status									
Negative	72 (31.3%)	11 (23.4%)	*p*=0.281	25 (26.9%)	4 (22.2%)	*p*=0.680	47 (34.3%)	7 (24.1%)	*p*=0.288
Positive	158 (68.7%)	36 (76.6%)		68 (73.1%)	14 (77.8%)		90 (65.7%)	22 (75.9%)	
Distant metastases									
Negative	113 (49.1%)	19 (40.4%)	*p*=0.276	45 (48.4%)	9 (50.0%)	*p*=0.900	68 (49.6%)	10 (34.5%)	*p*=0.137
Positive	117 (50.9%)	28 (59.6%)		48 (51.6%)	9 (50.0%)		69 (50.4%)	19 (65.5%)	
Cell differentiation									
Well/ Moderate	203 (88.3%)	41 (87.2%)	*p*=0.843	73 (78.5%)	15 (83.3%)	*p*=0.643	130 (94.9%)	26 (89.7%)	*p*=0.282
Poor	27 (11.7%)	6 (12.8%)		20 (21.5%)	3 (16.7%)		7 (5.1%)	3 (10.3%)	

Abbreviation: EGFR, epidermal growth factor receptor.

Table S5. ALFA allele frequencies of Visfatin SNPs

Visfatin SNPs	rs11977021		rs61330082		rs2110385		rs4730153	
**Population**	**Ref Allele**	**Alt Allele**	**Ref Allele**	**Alt Allele**	**Ref Allele**	**Alt Allele**	**Ref Allele**	**Alt Allele**
**Total**	C=0.751497	T=0.248503	G=0.78939	A=0.21061	T=0.63197	A=0.00000, G=0.36803	A=0.49024	G=0.50976
**Asian**	C=0.4625	T=0.5375	G=0.507	A=0.493	T=0.2	A=0.0, G=0.8	A=0.105	G=0.895
**South Asian**	C=0.716	T=0.284	G=0.717	A=0.283	T=0.82	A=0.00, G=0.18	A=0.37	G=0.63
**European**	C=0.755595	T=0.244405	G=0.75706	A=0.24294	T=0.5107	A=0.0000, G=0.4893	A=0.42751	G=0.57249
**Latin American 1**	C=0.7428	T=0.2572	G=0.671	A=0.329	T=1.00	A=0.00, G=0.00	A=0.435	G=0.565
**Latin American 2**	C=0.6862	T=0.3138	G=0.677	A=0.323	T=1.00	A=0.00, G=0.00	A=0.299	G=0.701
**African**	C=0.8940	T=0.1060	G=0.8920	A=0.1080	T=0.8547	A=0.0000, G=0.1453	A=0.7868	G=0.2132
**Other**	C=0.7309	T=0.2691	G=0.773	A=0.227	T=0.614	A=0.000, G=0.386	A=0.4687	G=0.5313

The **Al**lele **F**requency **A**ggregator (ALFA) analysis of the aggregate allele frequency of *visfatin* SNPs using the database of Genotypes and Phenotypes (dbGaP). Release Version: 20201027095038.

Figure S1

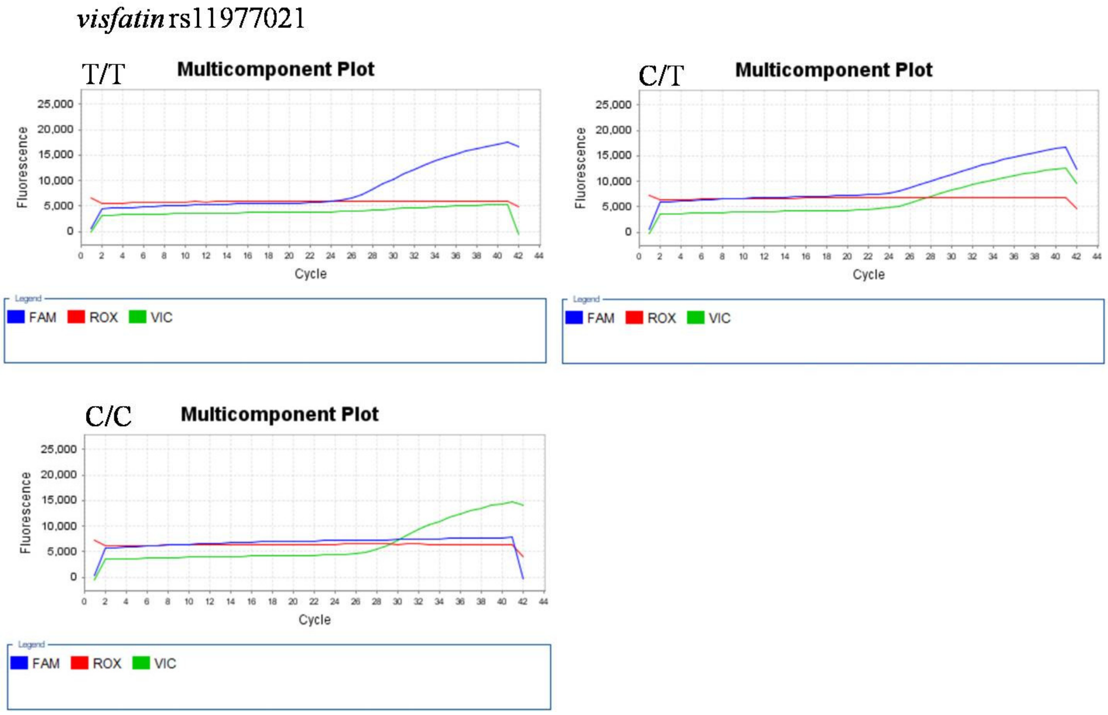

Figure S1: Representative TaqMan assay for *visfatin* rs11977021 genotyping.

Figure S2

visfatin rs61330082

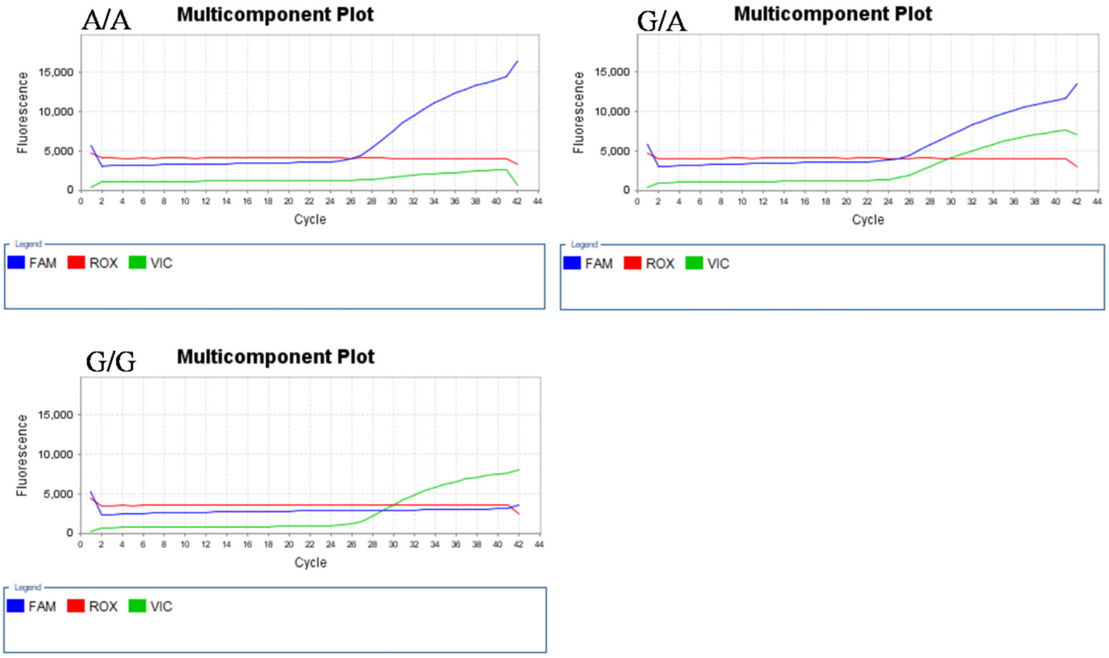

Figure S2: Representative TaqMan assay for *visfatin* rs61330082 genotyping.

Figure S3

visfatin rs2110385

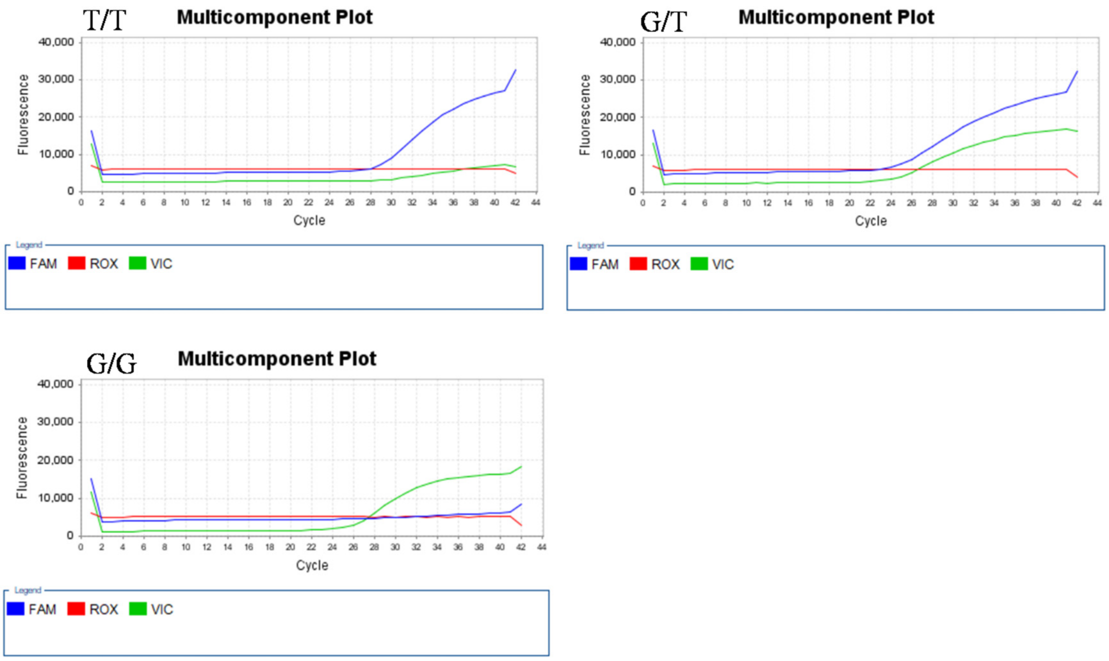

Figure S3: Representative TaqMan assay for *visfatin* rs2110385 genotyping.

Figure S4

visfatin rs4730153

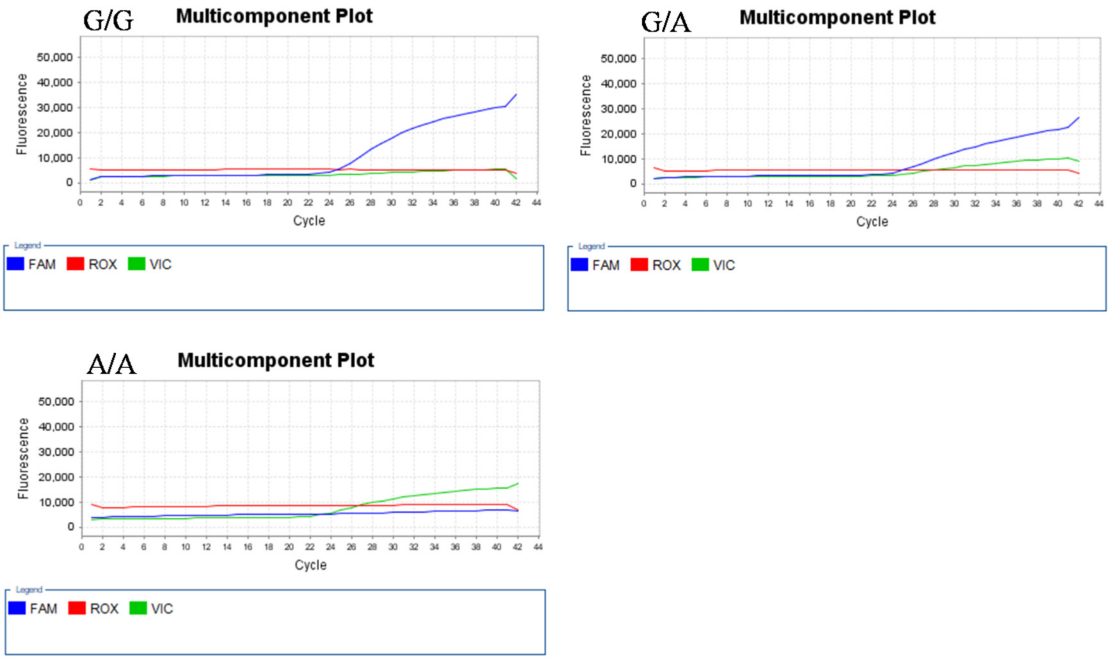

Figure S4: Representative TaqMan assay for *visfatin* rs4730153 genotyping.
